# Electroacupuncture Improves Microglial Polarization Induced‐Inflammation by Regulating the TGF‐β/Smad‐3 Signaling Pathway in Ischemic Stroke Mice

**DOI:** 10.1111/cns.70567

**Published:** 2025-08-20

**Authors:** Guoqiang Yang, Liulu Zhang, Yanlin Yuan, Maryam Mazhar, Dechou Zhang, Yong Liu, Guiquan Chen, Xuehui Fan

**Affiliations:** ^1^ Acupuncture and Rehabilitation Department, The Affiliated Traditional Chinese Medicine Hospital Southwest Medical University Luzhou Sichuan China; ^2^ Department of Magnetic Resonance Imaging, The Affiliated Traditional Chinese Medicine Hospital Southwest Medical University Luzhou Sichuan China; ^3^ School of Physical Education Southwest Medical University Luzhou Sichuan China; ^4^ National Traditional Chinese Medicine Clinical Research Base and Drug Research Center of the Affiliated Traditional Chinese Medicine Hospital Southwest Medical University Luzhou Sichuan China; ^5^ Department of Neurology, The Affiliated Traditional Chinese Medicine Hospital Southwest Medical University Luzhou Sichuan China; ^6^ Key Laboratory of Medical Electrophysiology, Ministry of Education and Medical Electrophysiological Key Laboratory of Sichuan Province, Collaborative Innovation Center for Prevention of Cardiovascular Diseases, Institute of Cardiovascular Research, Department of Cardiology, The Affiliated Hospital of Southwest Medical University Southwest Medical University Luzhou Sichuan China

**Keywords:** electroacupuncture, ischemic stroke, microglia, TGF‐β/Smad‐3 signaling

## Abstract

**Aims:**

This study aimed to investigate the mechanisms underlying the therapeutic effects of electroacupuncture (EA) at the Dazhui (GV14) and Baihui (GV20) acupoints in the treatment of ischemic stroke (IS).

**Methods and Results:**

The therapeutic efficacy of EA was evaluated using a middle cerebral artery occlusion (MCAO) mouse model. Neurological function was assessed through behavioral assessments, and infarct volume was measured using magnetic resonance imaging. Techniques such as immunofluorescence and western blotting were employed to analyze neural injury recovery, neuroinflammation, microglia/macrophage activation and polarization, as well as alterations in the TGF‐β/Smad3 signaling pathway. Our findings demonstrated that EA significantly improved neurological function and reduced infarct volume in MCAO mice. Furthermore, EA attenuated neuroinflammation by suppressing the polarization of microglia toward the pro‐inflammatory M1 phenotype. Additionally, EA decreased the expression of TGF‐β and Smad3 proteins following MCAO.

**Conclusion:**

EA may inhibit the M1 polarization of microglia/macrophages and provide a protective effect against ischemic brain injury by modulating the TGF‐β/Smad‐3 signaling pathway. These findings suggest that EA could be a potential therapeutic strategy for IS treatment.

## Graphical abstract

Schematic representation of the effect of EA on microglial polarization induced inflammation by regulating the TGF‐β/Smad‐3 signaling pathway in ischemic stroke mice. The therapeutic efficacy of electroacupuncture (EA) at Dazhui (GV14) and Baihui (GV20) acupoints in MCAO mice has been shown to activate the TGF‐β/Smad3 signaling pathway, modulating microglial polarization and reducing the inflammatory response. This mechanism contributes significantly to poststroke recovery, highlighting EA's potential as an effective treatment for IS.

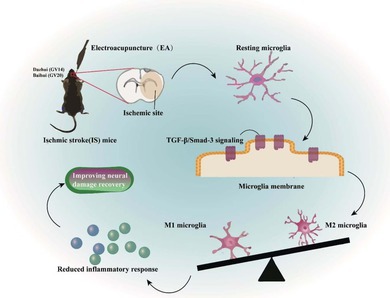

## 1 Introduction

Stroke is a leading cause of central nervous system (CNS) disorders, significantly contributing to increased mortality and morbidity, particularly in the aging population. Ischemic stroke (IS), the most prevalent type, accounts for approximately 80% of all stroke cases, affecting an estimated 24.9 million people annually [1, 2]. However, the complex pathogenesis of IS, coupled with substantial individual variability in treatment responses, presents substantial challenges for timely and effective intervention. Currently, safe and reliable therapeutic options for IS remain limited.

Neuroinflammation has emerged as a critical concern in IS therapy, as it exacerbates neurological dysfunction and impedes recovery [3]. It is a key pathological component of IS, occurring at nearly all stages of the disease [4]. Following cerebral occlusion, the affected brain region experiences glucose and oxygen deprivation, leading to neuronal damage. A hallmark of IS‐induced neuroinflammation is the accumulation of self‐renewed, polarized microglia in the penumbra, which drives secondary demyelination and neuronal loss [5]. This process involves the rapid activation of microglia, which migrate to the injury site in response to neurotransmitters, free radicals, and other injury‐associated signals. The release of pro‐inflammatory mediators during this activation serves as a biomarker of neuroinflammation [6, 7]. Microglial activation is evident across the acute, subacute, and chronic stages of IS and correlates with the severity of ischemia [8].

Activated microglia polarize into two distinct phenotypes: M1 and M2. M1 microglia produce pro‐inflammatory cytokines and cytotoxic substances, such as TNF‐α, IL‐6, and IL‐1β, which exacerbate neuroinflammation, disrupt the blood–brain barrier (BBB), and worsen brain injury [8, 9]. Conversely, M2 microglia secrete anti‐inflammatory cytokines and growth factors, including Arg‐1, CD206, IL‐10, and transforming growth factor‐β (TGF‐β), promoting angiogenesis and synaptic remodeling [10, 11]. In addition to microglia, peripheral macrophages infiltrate the brain through the compromised BBB, accumulating at the ischemic site and contributing to the inflammatory milieu [12].

The TGF‐β/Smad3 signaling pathway is implicated in numerous pathological processes associated with brain disorders, including stroke, traumatic brain injury, and Parkinson's disease [13, 14, 15]. This pathway plays a pivotal role in regulating cellular processes such as immune responses, growth, differentiation, and apoptosis. Dysregulation of TGF‐β/Smad3 signaling has been linked to autoimmune diseases and chronic inflammatory conditions due to its critical role in immune modulation [15]. Understanding the complex roles of the TGF‐β/Smad3 pathway in health and disease offers valuable insights and identifies potential therapeutic targets for a range of neurological and systemic conditions.

Electroacupuncture (EA) at the Baihui (GV20) and Dazhui (GV14) acupoints has been shown to rescue the impaired blood perfusion and neuronal activity in the contralateral primary motor cortex and the contralateral primary sensory cortex induced by stroke, and alleviation of depressive symptoms [16, 17, 18]. EA confers neuroprotection against brain damage following cerebral ischemia–reperfusion injury [19, 20, 21]. This study aims to investigate the therapeutic potential of EA at the Dazhui (GV14) and Baihui (GV20) acupoints in the treatment of IS. Specifically, we examine whether EA can modulate microglial polarization to alleviate neuroinflammation in IS mouse models. Additionally, we explore the potential link between EA‐induced regulation of microglial polarization and the TGF‐β/Smad3 signaling pathway in microglia.

## 2 Materials and Methods

### 2.1 Animals

**FIGURE 1:**
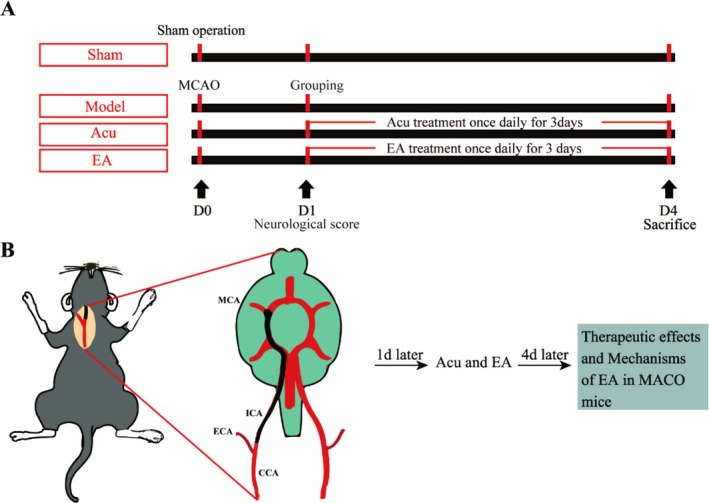
The experimental protocols. (A) Overview of group assignments, treatment procedures, and a detailed timeline for the experimental mice. (B) Schematic representation of MCAO‐induced mice and related treatments at the following 3 days.

Forty male wild‐type C57BL/6 mice (8–9 weeks old, weighing 22–25 g) were purchased from Chongqing Tengxin Biotechnology Co. Ltd. (Chongqing, China). All mice were housed under standardized conditions, including a controlled temperature (23°C ± 2°C), a 12/12‐h light/dark cycle, relative humidity (65% ± 5%), and free access to food and water. The study was conducted in accordance with the “Guidance Suggestions for the Care and Use of Laboratory Animals” issued by the Ministry of Science and Technology of China. The experimental protocol was approved by the Animal Ethical Committee of the Animal Center of Southwest Medical University (Approval No. 20240920‐025). Efforts were made to minimize the number of animals used and to reduce pain during experimental procedures. The mice were randomly divided into four groups: the sham group (Sham), the model group (Model), the acupuncture group (Acu), and the electroacupuncture group (EA) (Figure 1A).

### 2.2 MCAO Surgery

The mice were anesthetized with 40 mg/kg of 1% pentobarbital sodium and subjected to a middle cerebral artery occlusion (MCAO) procedure. This was performed by inserting a filament (Doccol, USA) through the right internal carotid artery (ICA) to induce ischemia. The method for establishing the mouse MCAO model was shown in the schematic diagrams (Figure 1B). After 30 min, the filament was carefully removed to allow reperfusion. During both the surgery and the postoperative recovery period, the mice were maintained at a constant body temperature of 37°C until they regained consciousness.

### 2.3 EA Stimulation at Acupoints

**FIGURE 2:**
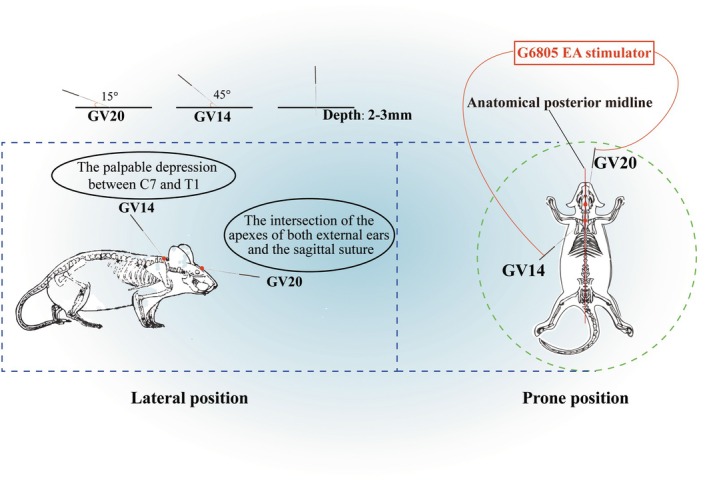
The localization for acupoints (GV20 and GV14) in mice and specific anatomical landmarks.

EA stimulation was conducted as described in our previous study [22]. EA stimulation was applied for 20 min, starting 24 h postreperfusion. Two sterile acupuncture needles (0.25 mm diameter) were administered at the Dazhui (GV14) and Baihui (GV20) acupoints following standardized rodent anatomical guidelines (Figure 2). GV14 localization: Positioned on the dorsal midline at the palpable depression between the spinous processes of the seventh cervical vertebra (C7) and the first thoracic vertebra (T1). This landmark corresponds to the cervicothoracic junction, identifiable by the prominent C7 spinous process in mice. GV20 localization: Located on the cranial dorsal midline at the intersection of the interaural line (connecting the apexes of both external ears) and the sagittal suture. This neuroanatomical landmark ensures consistent positioning across subjects. These needles in these acupoints were connected to a G‐6805 EA stimulator (HM6805, China). Additional EA sessions were administered at 24‐h intervals for up to four consecutive days.

### 2.4 Neurological Function Assessment by Modified Neurologic Severity Score (mNSS) Test and Behavioral Tests

The modified mNSS was applied as previously described to assess neurological deficits 24 h post‐IS (Table 1) [23]. The mNSS system evaluates motor, sensory, and reflex impairments, providing a comprehensive measure of IS‐induced neurological damage. Scores range from 0 (normal function) to 18 (maximum deficit), with higher scores indicating greater severity of neurological impairment: scores of 13–18 denote severe damage, 7–12 moderate damage, and 1–6 slight damage. In this system, one point is assigned for the inability to complete a task or the absence of specific reflexes, with higher scores reflecting more severe injury. In the present study, mice with moderate neurological deficits (mNSS scores of 7–12) were selected for subsequent experiments.

**TABLE 1:** mNSS test for neurological deficits.

Motor tests	
Raising by tail (normal = 0, maximum = 3)	(3)
Flexion of fore limb	1
Flexion of hind limb	1
Head moved > 10 to vertical axis within 30 s	1
Placing on floor (normal = 0; maximum = 3)	(3)
Normal walk	0
Inability to walk straight	1
Circling toward the paretic side	2
Falls down to paretic side	3
Sensory tests (normal = 0; maximum = 2)	(2)
Placing test (visual and tactile test)	1
Proprioceptive test (deep sensation, pushing paw against table edge to stimulate limb muscles)	1
Beam balance tests (normal = 0; maximum = 6)	(6)
Balances with steady posture	0
Grasps side of beam	1
Hugs beam and 1 limb falls down from beam	2
Hugs beam and 2 limbs fall down from beam, or spins on beam (> 60 s)	3
Attempts to balance on beam but falls off (> 40 s)	(4)
Attempts to balance on beam but falls off (> 20 s)	5
Falls off, no attempt to balance or hang on the beam (< 20 s)	6
Reflex absence and abnormal movements (normal = 0, maximum = 4)	4
Pinna reflex (head shaken when auditory meatus is touched)	1
Corneal reflex (eye blink when cornea is lightly touched with cotton)	1
Startle reflex (motor response to a brief noise from clapping hands)	1
Seizures, myoclonus, myodystony	1
Maximum points	(18)

*Note:* One point is given for an absent reflex tested or for the animal's inability to perform a task: 1–6 mild injury, 7–12 moderate injury, 13–18 severe injury.

For the beam balance test, mice were placed on a 2 cm‐wide beam and allowed to traverse it to reach a terminal home cage. The time required for each mouse to complete the crossing was recorded, with a maximum observation period of 1 min. Additionally, the number of paw slips during the traversal was documented, and overall performance was evaluated based on predefined scoring criteria. To ensure consistency, all mice were conditioned and trained on the balance beam 2 days prior to the IS induction, as detailed in our previous study (Table 2) [23, 24]. To minimize observer bias, all behavioral assessments were conducted by two independent investigators blinded to treatment allocation. Mice were randomly coded with nonsequential alphanumeric identifiers prior to testing, and group assignments remained concealed until final data analysis. Between‐group variance was calculated using one‐way ANOVA, whereas within‐group variance was quantified by standard deviation and 95% confidence intervals across repeated trials.

**TABLE 2:** Neurological scoring scale for beam walking test.

Score	Performance on the beam
7	Traverses beam normally with both affected paws on horizontal beam surface, neither paw ever grasps the side surface, and there are no more than two paw slips; toe placement style is the same as preinjury.
6	Traverses beam successfully and uses affected limbs to aid > 50% of steps along beam.
5	Traverses beam successfully but uses affected limbs in < 50% of steps along beam.
4	Traverses beam and, at least once, places affected limbs on horizontal beam surface.
3	Traverses beam by dragging affected hind limbs.
2	Unable to traverse beam but places affected limbs on horizontal beam surface and maintains balance for ≥ 5 s.
1	Unable to traverse beam; cannot place affected limbs on horizontal beam surface.

### 2.5 Brain Water Content

Brain water content was determined utilizing a method previously described [22]. Briefly, animals were anesthetized and subsequently decapitated. Brain samples were quickly extracted and immediately weighed using a precise electronic balance to determine the wet weight. The samples were subsequently dried in a thermostat set at 100°C for 24 h, after which they were reweighed to determine the dry weight. Brain water content was calculated by the formula: [(wet weight − dry weight)/wet weight] × 100%.

### 2.6 Magnetic Resonance Imaging (MRI) in MCAO Mice Following EA

For the MRI examination, mice were administered 40 mg/kg of 1% pentobarbital sodium for anesthesia. MRI scans were conducted on a 3‐Tesla system (Siemens, Germany) at the Affiliated Traditional Chinese Medicine Hospital of Southwest Medical University. A T1‐weighted fast spin‐echo sequence was employed with the following parameters: repetition time/echo time = 500/11 ms, a field of view of 16 × 16 mm^2^, a matrix resolution of 256 × 256, and a slice thickness of 0.75 mm for both coronal and axial planes. The resulting T1‐weighted images were saved in 1019 × 602‐pixel resolution files for the evaluation of lesions.

### 2.7 Immunofluorescence Staining

Brain slices were prepared as described previously [25] and permeabilized with 0.3% Triton X‐100 for 15 min. Subsequently, the slices were blocked with 5% BSA for 1 h and incubated overnight at 4°C with the following primary antibodies: rabbit anti‐GFAP (Proteintech, 1:100 dilution), rabbit anti‐GALC (Proteintech, 1:100 dilution), rabbit anti‐NeuN (CST, 1:100 dilution), mouse anti‐Iba1 (Abcam, diluted 1:100), rabbit anti‐CD80 (Proteintech, 1:100 dilution), rabbit anti‐CD86 (Proteintech, 1:100 dilution), rabbit anti‐Arg‐1 (Proteintech, 1:100 dilution), and rabbit anti‐TGF‐β (Proteintech, 1:100 dilution). After PBS washes, secondary antibodies were applied and incubated at room temperature (RT) for 2 h. The secondary antibodies used included Alexa FluorTM 555 goat anti‐rabbit IgG (Invitrogen, diluted 1:100), Alexa Fluor 555 goat anti‐mouse IgG (Life Technologies, 1:100 dilution), Alexa Fluor 488 goat anti‐rabbit IgG (Invitrogen, 1:100 dilution), and Alexa FluorTM 488 conjugated anti‐mouse IgG (Invitrogen, 1:100 dilution). Nuclei were counterstained with DAPI. Images were captured using a Leica DM4B orthotopic fluorescence microscope (Leica, Germany) and analyzed using ImageJ software.

### 2.8 Western Blotting

Protein expression levels were analyzed by Western blotting. The primary antibodies used were rabbit anti‐GFAP antibody (Proteintech, 1:1000 dilution), rabbit anti‐GALC antibody (Proteintech, 1:1000 dilution), rabbit anti‐NeuN antibody (CST, 1:1000 dilution), rabbit anti‐IL‐6 antibody (Proteintech, 1:1000 dilution), rabbit anti‐IL‐1β antibody (Abcam, 1:1000 dilution), rabbit anti‐CD80 antibody (Proteintech, 1:1000 dilution), rabbit anti‐CD86 antibody (Proteintech, 1:1000 dilution), rabbit anti‐Arg‐1 antibody (Proteintech, 1:1000 dilution), rabbit anti‐TGF‐β antibody (Proteintech, 1:1000 dilution), rabbit anti‐p‐Smad3 antibody, and rabbit anti‐Smad3 antibody (Abcam, 1:1000 dilution). Membranes were incubated with these antibodies overnight at 4°C. After washing, the membranes were incubated with HRP‐conjugated donkey anti‐rabbit or mouse IgG antibodies (Thermo Fisher, 1:3000 dilution) at RT for 1 h. Protein bands were visualized using enhanced chemiluminescence (ECL) reagent (Vazyme, China) and captured by an ImageQuant ECL Imager. Relative protein expression levels were quantified by normalizing band intensities to GAPDH or total Smad3 using ImageJ software.

### 2.9 Statistical Analysis

Statistical analyses were performed using GraphPad Prism 8 software. The normality of the data distribution was assessed using the Shapiro–Wilk test. Quantitative data were expressed as mean ± SEM. The significance of differences among multiple groups were evaluated using one‐way analysis of variance (ANOVA) followed by post hoc comparisons using Tukey's multiple comparison tests, with significance set at *p* < 0.05.

## 3 Results

### 3.1 EA Ameliorated Neurological Functional Deficits and Reduced Neural Damage in MCAO Mice

**FIGURE 3:**
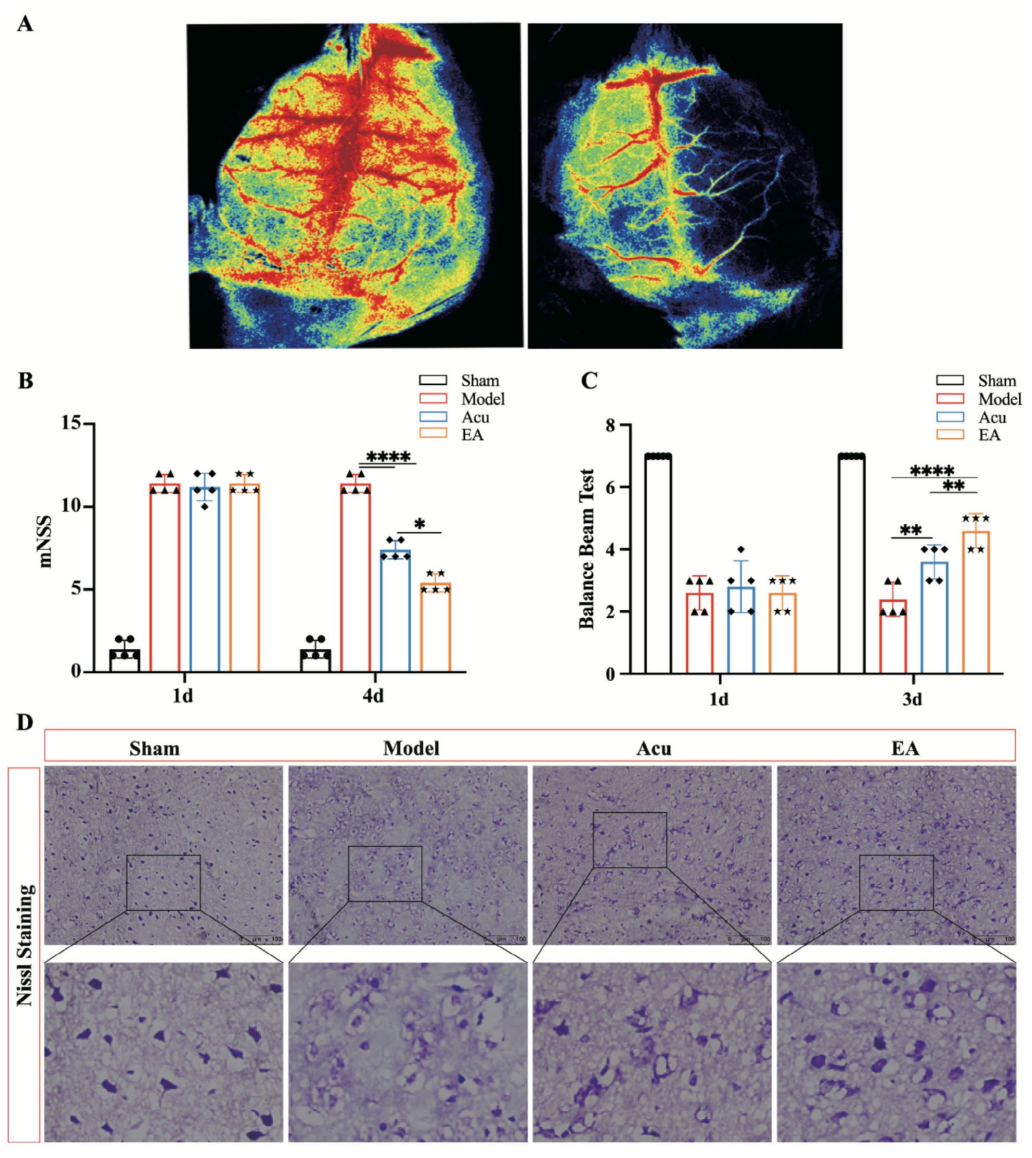
EA ameliorated neurological functional deficits and reduced neural damage in the infarction area in MCAO mice. (A) Laser Speckle Imaging confirms the successful establishment of the MCAO model. (B, C) Neurological deficits significantly decreased on the fourth day postreperfusion following EA treatment. (D) Nissl staining demonstrates that EA notably alleviated neuronal damage in the infarction area, showing improved neuronal morphology and restoration of Nissl bodies. *p < 0.05; **p < 0.01; ****p < 0.0001; *n* = 5 for each group. Scale bar = 100 μm.

The MCAO mouse model was successfully established, and EA treatment effectively alleviated neurological deficits and neural damage (Figure 3). Laser Speckle Imaging (LSI) confirms the successful establishment of the MCAO model by evaluating the blood perfusion (Figure 3A). Cerebral ischemia was assessed via LSI, suggesting that the ischemic region exhibited a complete absence of detectable blood flow. These hemodynamic findings confirm successful MCAO and establish a validated model of focal cerebral ischemia. Neurological deficit scores significantly increased following MCAO but were notably improved by Acu and EA treatments by the fourth day (Figure 3B,C). Importantly, EA treatment showed a more pronounced reduction in neurological functional deficits compared to Acu. Nissl staining demonstrated distinct neuronal damage in the right hemispheric cortical and striatal regions within the ischemic territory following MCAO induction. These neurons exhibited significantly reduced Nissl bodies, light blue cytoplasmic staining, and prominent vacuolation, indicating intracellular structural destruction and liquefaction (Figure 3D). Both Acu and EA treatments mitigated these changes, as evidenced by increased Nissl bodies, darker cytoplasmic staining, restored neuronal volume, and reduced cytoplasmic vacuoles. Furthermore, EA treatment demonstrated superior restorative effects compared to Acu, suggesting its enhanced efficacy in promoting neural recovery.

### 3.2 EA Ameliorated Infarct Area and Brain Water Content in MCAO Mice

**FIGURE 4:**
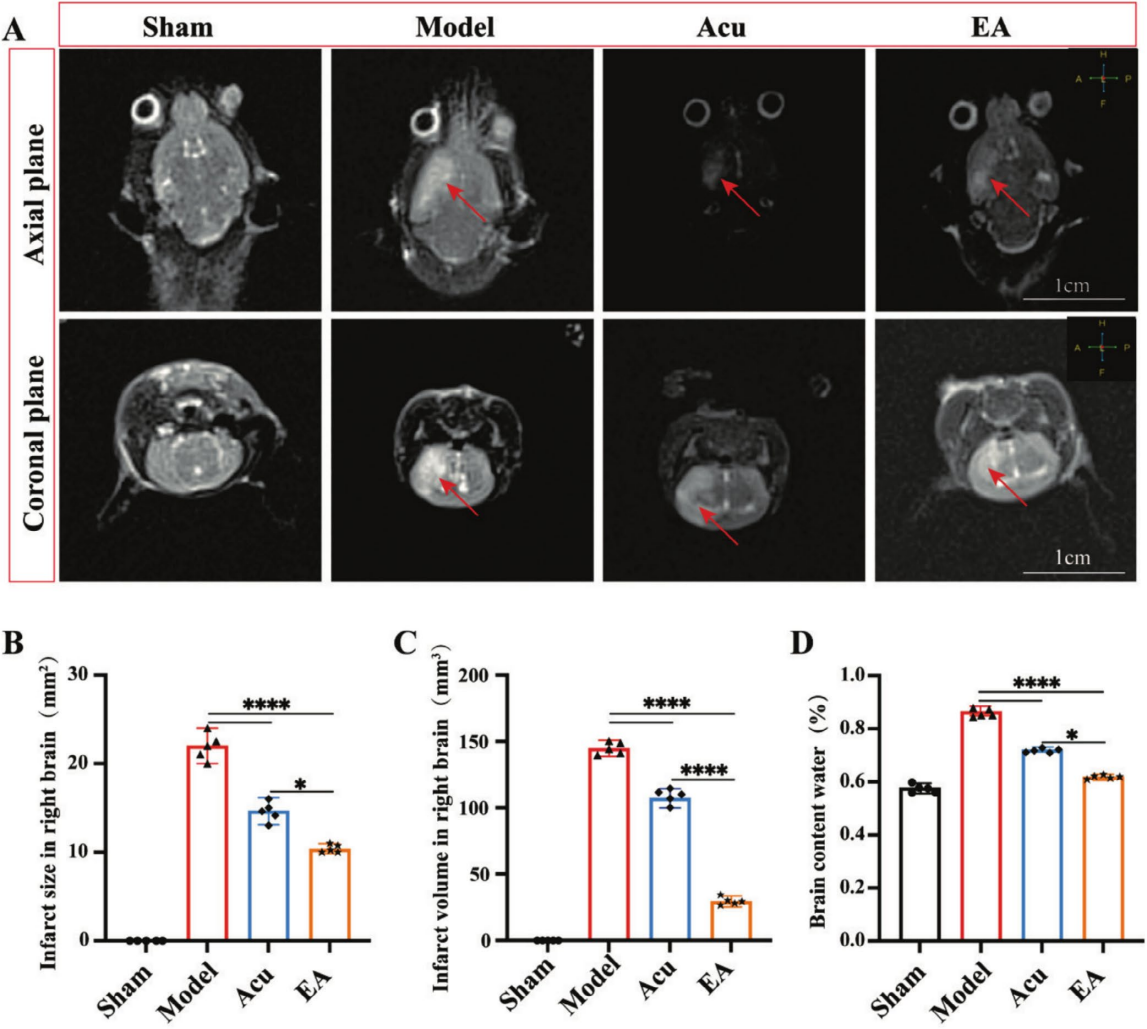
Infarct area and brain water content in different groups assessed by 3.0T MRI and water content analysis. (A–C) Infarct size and volume measured using MRI, and the highlighted regions indicate infarct areas in the right hemisphere. Scale bar = 1 cm. (C) Brain water content measurement. *p < 0.05; ****p < 0.0001; *n* = 5 per group.

To investigate the effects of EA on infarct area and brain water content, we employed T1‐weighted MRI. T1‐weighted MRI images show an apparent infarct (highlighted area) in the right hemisphere of the model group in both coronal and axial planes, compared to the sham group. Both Acu and EA treatments significantly reduced the infarct area and volume, with EA demonstrating a more pronounced reduction compared to Acu (Figure 4A–C). Brain water content increased significantly after MCAO. Both Acu and EA treatments effectively reduced brain water content, with EA showing a greater alleviation compared to Acu (Figure 4D).

### 3.3 EA Increased the Protein Expression of NeuN and GALC Decreased the Protein Level of GFAP in IS Mice

**FIGURE 5:**
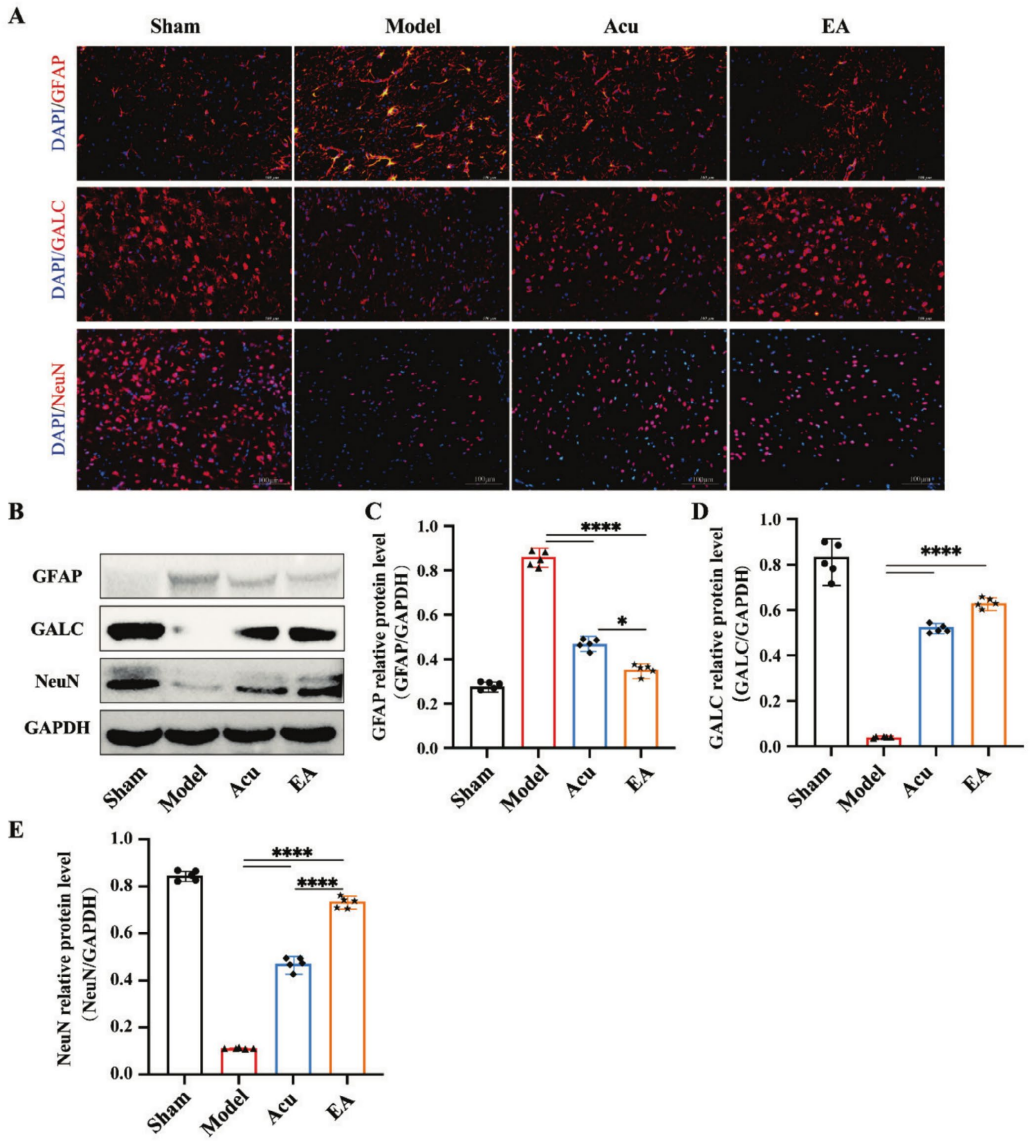
EA treatment increased NeuN and GALC protein expression and decreased GFAP protein expression in IS mice. (A) Immunofluorescent results showing NeuN, GALC, and GFAP‐positive cells in the ipsilateral striatum of IS mice brain tissue after MCAO (*n* = 5 for each group). Scale bar = 100 μm. (B–E) Representative bands and mean values of western blot analyzed GFAP, GALC, and NeuN protein expression levels. *n* = 5 for each group; *p < 0.05; ****p < 0.0001.

Immunofluorescence and western blotting were performed to assess the analysis of NeuN, GALC, and GFAP protein expression in the ipsilateral striatum of mice brain tissue across different groups. Specifically, Figure 5A showed the immunofluorescence staining of NeuN, GALC, and GFAP in brain tissue. After IS, the expression levels of NeuN and GALC increased, while GFAP expression decreased. In both treatment groups, positive staining for NeuN and GALC was higher, and GFAP staining was reduced (Figure 5B–E). Following MCAO, NeuN and GALC expression levels were lower, and GFAP expression was higher compared to the sham group. After treatment, NeuN and GALC protein levels increased, while GFAP expression decreased. EA treatment significantly enhanced NeuN and GALC levels and reduced GFAP expression.

### 3.4 EA Downregulated Microglial Polarization‐Associated Neuroinflammation by Regulating the TGF‐β/Smad‐3 Signaling Pathway in MACO Mice Following IS

**FIGURE 6:**
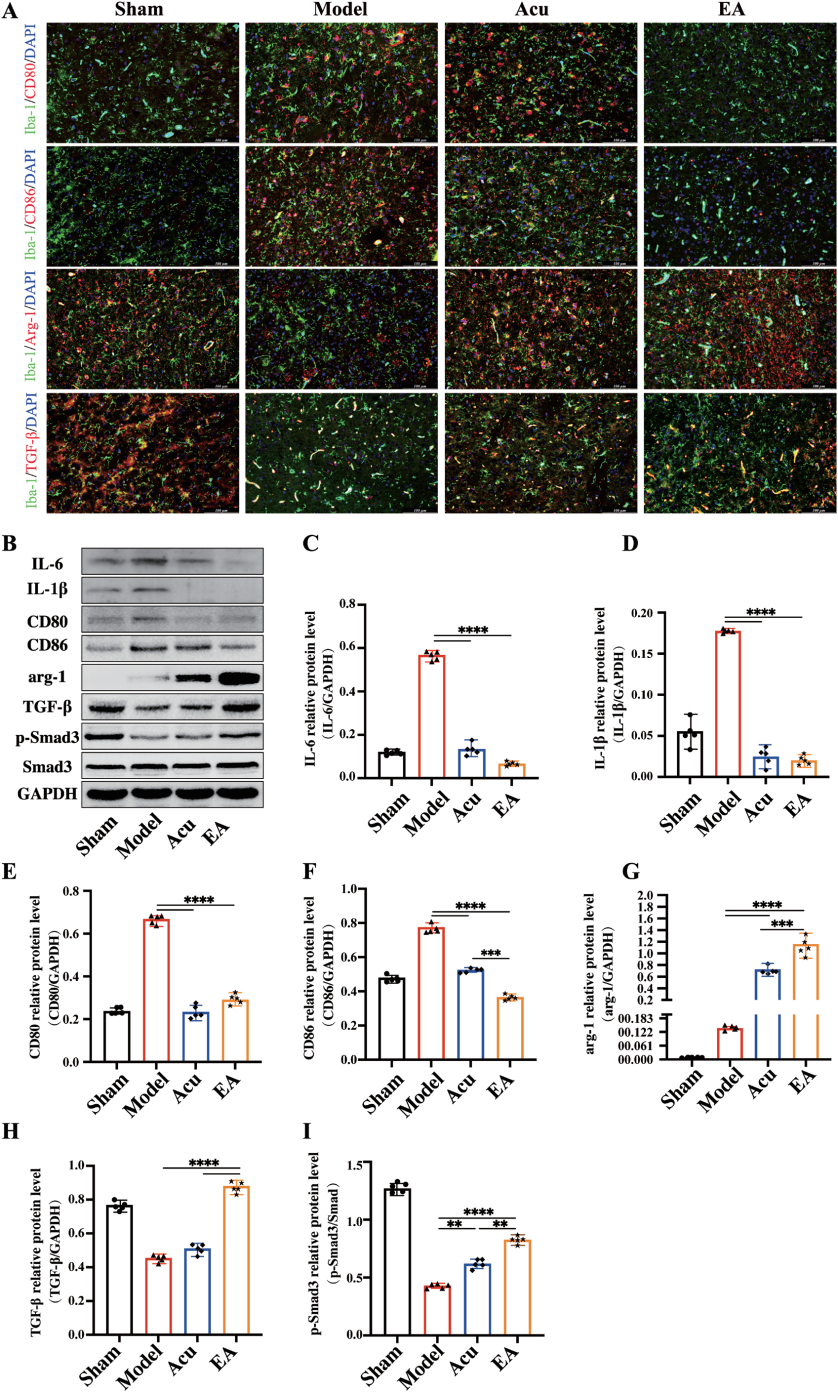
EA improved microglial polarization associated with neuroinflammation through the TGF‐β/Smad3 signaling pathway in MACO mice. (A) Immunofluorescence for the microglial polarization proteins in the ischemic cerebral cortex. (B–I) Western blotting results of the inflammatory factors, microglial polarization proteins, TGF‐β, and Smad3. **p < 0.01; ***p < 0.001; ****p < 0.0001; *n* = 5 for each group.

Co‐labeling immunofluorescence of Iba1 (a marker of microglia) with CD80, CD86 (markers of M1 microglia), Arg‐1, and TGF‐β (markers of M2 microglia) showed a significant increase in the number of Iba1^+^ cells coexpressing CD80^+^ and CD86^+^ markers (indicative of M1 microglia) and a marked decrease in the number of Iba1^+^ cells with Arg‐1^+^ and TGF‐β^+^ markers (indicative of M2 microglia) in the MCAO group. These effects were significantly reversed by EA treatment when compared to Acu therapy (Figure 6A). Figure 6B–I presented the protein expression levels and analyses of neuroinflammation, microglial polarization, and the TGF‐β/Smad3 signaling pathway as measured by western blotting. The results demonstrated that EA treatment significantly downregulated the expression of pro‐inflammatory markers IL‐6 and IL‐1β and improved M2 microglial polarization compared to Acu treatment, which was consistent with the immunofluorescence findings. Additionally, western blotting revealed a significant decrease in the protein expression levels of TGF‐β and Smad3 following MCAO. These data suggested that both Acu and EA treatments enhanced the expression of TGF‐β and Smad3, with EA treatment resulting in a significant upregulation of TGF‐β and Smad3 compared to Acu therapy.

## 4 Discussion

EA is a widely recognized and effective therapy used in stroke rehabilitation, which involves stimulating specific acupuncture points with electrical currents. This method promotes recovery through various mechanisms, including pain relief, anti‐inflammatory effects, and enhanced blood circulation, all of which contribute to overall stroke rehabilitation [26]. The stimulation of GV14 and GV20 acupoints has been well established in stroke treatment, with significant efficacy demonstrated in both clinical and preclinical studies [22, 27, 28]. In our study, EA at these acupoints reduced cerebral infarction volume and improved behavioral performance in MCAO mice. Moreover, we found that EA treatment downregulated microglial polarization‐associated neuroinflammation by regulating the TGF‐β/Smad‐3 signaling pathway, highlighting a potential therapeutic mechanism of EA in stroke recovery.

Brain edema, an early and devastating complication of IS, critically contributes to poor clinical outcomes [29, 30]. Previous studies have demonstrated that cerebral autoregulation impairment exacerbates brain edema and neurological deterioration, and aberrant cerebral autoregulation will worsen cerebral perfusion [31]. In this study, both Acu and EA treatments reduced brain water content in the right ischemic hemisphere of MCAO mice, with EA treatment inducing a more pronounced reduction compared to Acu therapy. MRI findings further corroborated the superior regulatory effect of EA on brain edema mitigation.

Following IS, extensive glial and neuronal cell death are induced through the mechanisms, such as neuroinflammation, oxidative stress, and cytotoxic signaling [32, 33, 34, 35]. Oligodendrocyte loss correlates with white matter demyelination, while neuronal apoptosis exacerbates functional neurological deficits. Reactive astrogliosis, a hallmark of CNS injury, initially exerts neuroprotective effects via neurotrophic factor secretion to preserve neuronal viability. However, persistent astrocyte activation culminates in fibrotic glial scar formation, creating a physical and biochemical barrier that critically inhibits axonal regeneration and synaptic reconnection during later recovery phases [36, 37]. This study demonstrated reduced NeuN (neuronal marker) and GALC (oligodendrocyte marker forming myelination) protein expression, alongside elevated GFAP (astrocytic marker) levels in the ischemic brain regions of MCAO mice. Compared to Acu therapy, EA treatment significantly upregulated NeuN and GALC expression while suppressing GFAP overexpression. These findings collectively suggest that EA enhances neuronal survival, promotes myelination, and mitigates astroglial scar formation, thereby alleviating neurological deficits.

After IS, microglia become activated, and infiltrating macrophages migrate to the injury site, differentiating into two distinct phenotypes: the pro‐inflammatory M1 phenotype and the anti‐inflammatory M2 phenotype. These phenotypes coexist and demonstrate mutual antagonism throughout the pathological progression of IS. Increased activation of M1 microglia/macrophages exacerbates neuroinflammation, whereas enhanced M2 microglia/macrophage activation can help alleviate stroke outcomes [23, 38]. The interaction between these two phenotypes critically influences the fate of injured neurons [39, 40, 41]. Our study found that EA significantly suppressed the polarization of the M1 phenotype while promoting the polarization of M2 microglia/macrophages. This shift toward M2 polarization suggests that EA restores the balance between the M1 and M2 phenotypes, thereby reducing inflammation and supporting brain repair. Elevated levels of M1 microglia contribute to neuronal damage through the secretion of pro‐inflammatory cytokines and neurotoxic chemokines [41]. Consistent with this, our results suggest that EA mitigated neuronal damage in the brain tissue following IS by modulating microglial polarization and associated neuroinflammation.

TGF‐β plays a key role in various biological processes, including inflammatory reactions, and is crucial in the CNS, where it is predominantly expressed in neurons and microglia [42]. As a pleiotropic cytokine, TGF‐β has been shown to exert neuroprotective effects through several mechanisms. It is involved in regulating synaptic growth and differentiation, neurotransmitter release, and the distribution and phosphorylation of synaptic proteins [43]. The Smad3 protein is a key transducer of TGF‐β signals, and studies suggest that Smad3 plays an essential role in regulating microglial function. Recent research has also highlighted the role of TGF‐β/Smad3 signaling in modulating GABA neurotransmission, particularly in the context of parkinsonism and cognitive alterations [15]. In our study, we found that EA treatment increased the protein levels of both TGF‐β and p‐Smad3, indicating that EA modulates the TGF‐β/Smad3 signaling pathway. This modulation likely contributes to the neuroprotective effects observed in the IS model, helping to mitigate neural damage during the IS process in MCAO mice.

In the context of pulmonary research, incubation of mouse lung microvascular endothelial cells with the specific TGF‐β inhibitor SB431542 modulated hyperoxia‐induced endothelial‐mesenchymal transition through regulating the TGF‐β/Smad3 pathway [44]. A study by Walsh et al. [45] demonstrated that the TGF‐β receptor antagonist SB431542 completely blocked TGF‐β‐induced Smad3 phosphorylation in IEC‐6 cells. Concurrently, another investigation revealed that enhanced TGF‐β activation significantly promoted Smad3 phosphorylation, further validating its role in downstream signaling. Notably, the TGF‐β inhibitor SB431542 has been shown to regulate neuroinflammation and apoptosis in both a traumatic brain injury rat model and an in vitro stretch‐injury model of rat neuronal cultures [46]. Additionally, Villapol et al. [47] reported that TGF‐β cytokine family signaling through Smad3 exerts early‐phase neuroprotection in the damaged cortex and hippocampus following injury. Collectively, these findings highlight the critical role of the TGF‐β/Smad3 signaling axis in inflammatory diseases. This also suggests that EA may improve neurological function following IS by regulating the TGF‐β/Smad3 signaling.

The present study has several limitations that warrant discussion. First, while upregulation of the TGF‐β/Smad3 pathway is clearly documented, the observed association with M1/M2 polarization remains correlational rather than causal. The increased expression of TGF‐β and Smad3 in our model does not establish a direct causal link to microglial polarization toward the M1 phenotype, necessitating further mechanistic investigations to elucidate the underlying signaling cascades. Second, the absence of pharmacological inhibition experiments (e.g., using a TGF‐β receptor blocker) precludes full mechanistic validation of the pathway. Given the promising role of TGF‐β/Smad3 signaling in IS, such experiments represent critical future directions. Third, the investigation of TGF‐β/Smad3 signaling was confined to the MCAO mouse model, which may limit the generalizability of our findings to other neurological diseases. This model‐specific constraint should be considered when extrapolating the results to discuss the pathway's role in broader pathological contexts. Fourth, the study lacked non‐acupoint control groups to evaluate the effects of non‐acupoint EA. We only compared EA at acupoints with the acupoint‐only (Acu) group, leaving the acupoint specificity of the observed effects undetermined. Notably, both clinical and preclinical studies have shown that EA at specific acupoints exerts significant therapeutic effects on neurogenic diseases compared to non‐acupoint stimulation [48, 49, 50]. These findings provide reliable evidence for acupoint specificity in CNS diseases, highlighting the need for such control groups in future research to validate this specificity within our experimental model.

In summary, our study highlights the therapeutic efficacy of EA at GV14 and GV20 acupoints in MCAO mice. We propose that the beneficial effects of EA in improving neural damage recovery following IS are mediated through the enhancement of the TGF‐β/Smad‐3 signaling pathway. These findings provide valuable mechanistic insights into how EA may promote recovery in stroke, supporting its potential clinical application as an adjunctive treatment for IS. Our results contribute to a better understanding of the underlying molecular mechanisms and offer a theoretical foundation for the use of EA in clinical stroke rehabilitation.

## Author Contributions

X.F. and G.Y. designed the experiments. X.F., G.Y., L.Z., Y.Y., and M.M. performed the experiments. X.F., G.Y., Y.Y., M.M., and D.Z. analyzed and interpreted the final data. X.F. and G.Y. wrote the manuscript. X.F., Y.L., and G.C. revised the manuscript. All authors have read and approved the final manuscript.

## Ethics Statement

This study was approved by the Animal Ethics Research Committee of Southwest Medical University, Luzhou, China (approval number: 20240920‐025; date of approval: 20.09.2024).

## Consent

The authors have nothing to report.

## Conflicts of Interest

The authors declare no conflicts of interest.

## Supporting information


**Appendix S1:** cns70567‐sup‐0001‐AppendixS1.pdf.

## Data Availability

The data that support the findings of this study are available from the corresponding author upon reasonable request.
