# Supplementary figures and images for: Electroacupuncture Improves Microglial Polarization Induced‐Inflammation by Regulating the TGF‐β/Smad‐3 Signaling Pathway in Ischemic Stroke Mice
Source: CNS Neurosci Ther. 2025 Aug 20;31(8):e70567. doi: 10.1111/cns.70567 (PMC12365391; doi:10.1111/cns.70567)

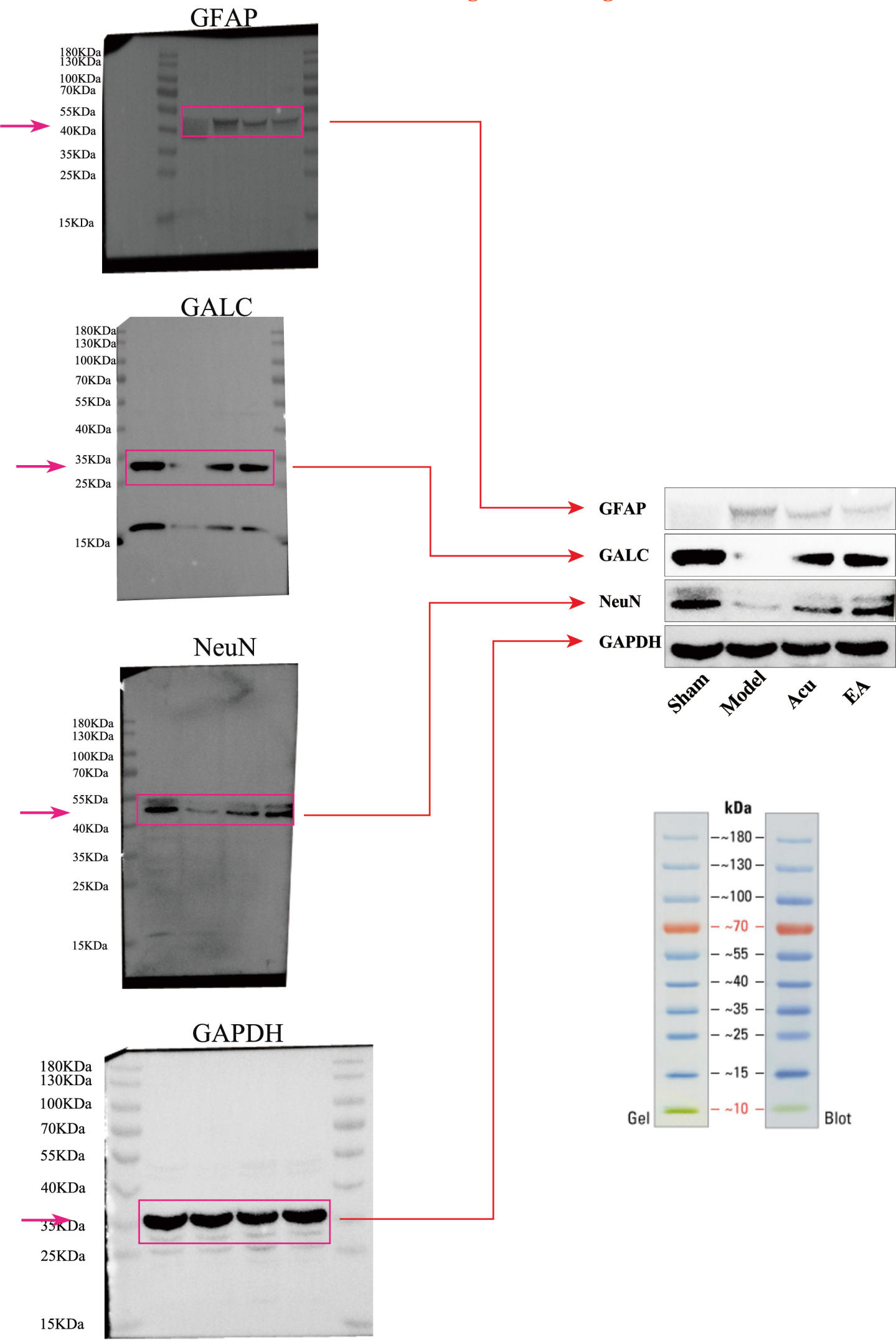

# Full unedited gel/blot for Figure 6

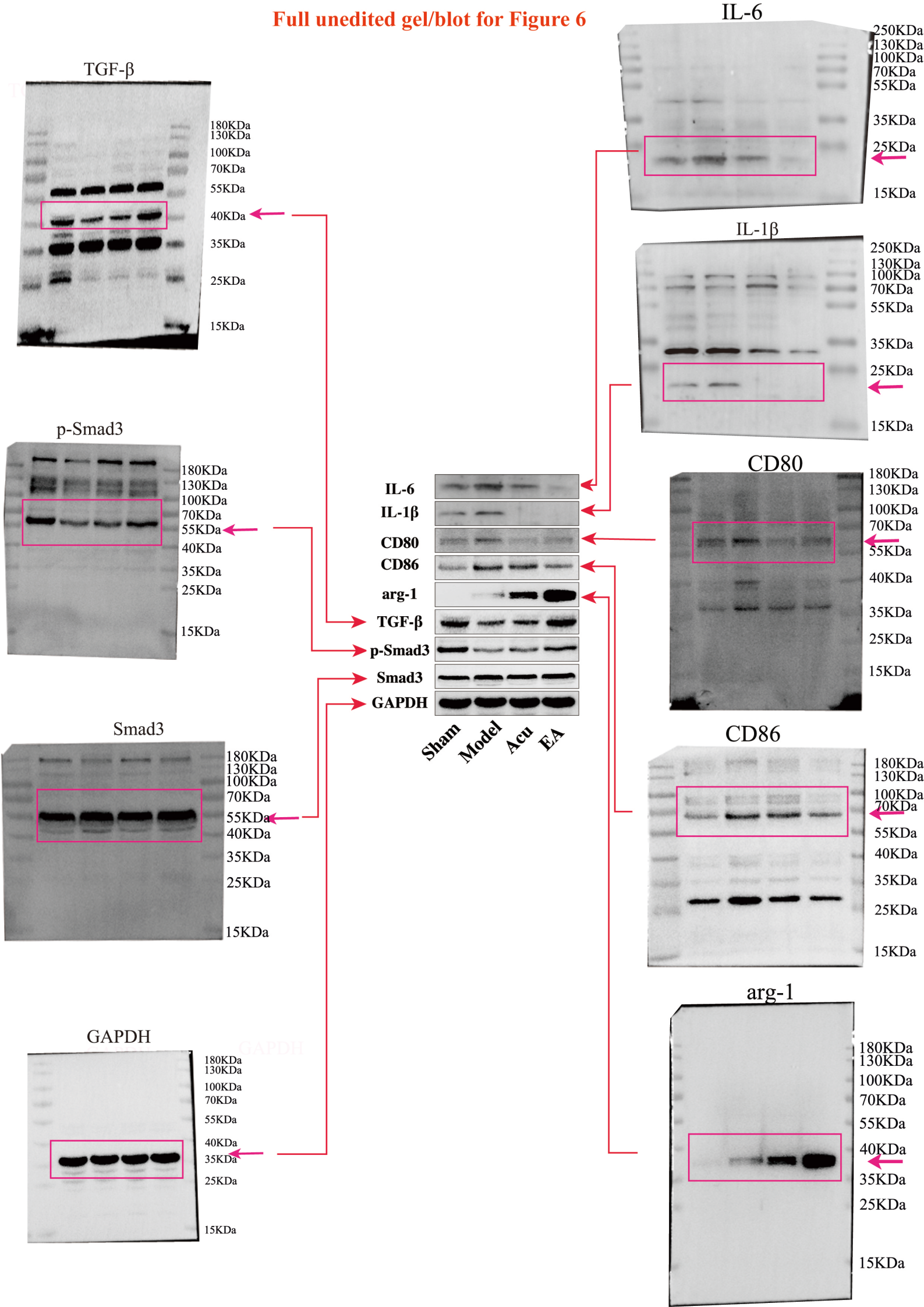

Supplement: Supplementary file 1 — Appendix S1: cns70567‐sup‐0001‐AppendixS1.pdf. [file CNS-31-e70567-s001.pdf]
